# Energy efficiency of respiration in mature and newborn reindeer

**DOI:** 10.1007/s00360-020-01284-3

**Published:** 2020-05-25

**Authors:** Simon Birger Byremo Solberg, Signe Kjelstrup, Elisa Magnanelli, Natalya Kizilova, Iratxe Lorea Casado Barroso, Mario Acquarone, Lars P. Folkow

**Affiliations:** 1 PoreLab, Department of Chemistry, Norwegian University of Science and Technology-NTNU, 7491 Trondheim, Norway; 2 SINTEF Energy Research, Sem Sælands vei 11, 7465 Trondheim, Norway; 3 Institute of Aeronautics and Applied Mechanics, Warsaw University of Technology, 00-665 Warsaw, Poland; 4 Department of Applied Mathematics, V. N. Karazin Kharkov National University, Kharkiv, 61022 Ukraine; 5 Present Address: Sea Turtle Conservation Project, Villa 198 Los Delfines, Paquera, Puntarenas, Costa Rica; 6 Department of Arctic and Marine Biology, University of Tromsø-the Arctic University of Norway, 9037 Tromsø, Norway

**Keywords:** *Rangifer tarandus*, Neonate, Respiration, Thermodynamics, Energy efficiency, Entropy production

## Abstract

Reindeer (*Rangifer tarandus*) have evolved elaborate nasal turbinate structures that are perfused via a complex vascular network. These are subject to thermoregulatory control, shifting between heat conservation and dissipation, according to the animal’s needs. The three-dimensional design of the turbinate structures is essential in the sense that they determine the efficiency with which heat and water are transferred between the structure and the respired air. The turbinates have already a relatively large surface area at birth, but the structures have yet not reached the complexity of the mature animal. The aim of this study was to elucidate the structure–function relationship of the heat exchange process. We have used morphometric and physiological data from newborn reindeer calves to construct a thermodynamic model for respiratory heat and water exchange and present novel results for the simulated respiratory energy losses of calves in the cold. While the mature reindeer effectively conserves heat and water through nasal counter-current heat exchange, the nose of the calf has not yet attained a similar efficiency. We speculate that this is probably related to structure-size limitations and more favourable climate conditions during early life. The fully developed structure–function relationship may serve as inspiration for engineering design. Simulations of different extents of mucosal vascularization suggest that the abundance and pattern of perfusion of veins in the reindeer nasal mucosa may contribute to the control of temperature profiles, such that nasal cavity tissue is sufficiently warm, but not excessively so, keeping heat dissipation within limits.

## Introduction

For most organisms, energy is a limited resource. Animal species, therefore, have evolved adaptations that reduce/minimize their energy needs. Homeotherms in cold habitats (e.g. at high latitudes) typically possess adaptations that restrict the loss of body heat to an environment that may be up to 100 °C colder than their deep body temperature. The range of mechanisms by which this may be achieved is impressive and has been described in many textbooks and reviews (e.g. Blix (2016); Hill et al. (2016)).

Reindeer (*Rangifer tarandus*) have a circumpolar distribution in the northern hemisphere and have successfully adapted to the arctic/subarctic environment, although population numbers have declined in recent years as a result of changing climatic conditions (Russell et al. 2018). Aside from carrying a fur of prime insulation quality (Moote 1955; Timisjärvi et al. 1984), reindeer may also restrict heat loss by physiological means, as recently reviewed by Blix (2016). One of the traits involves reduction of respiratory heat and water loss by means of nasal temporal counter-current heat exchange (Blix and Johnsen 1983; Blix 2016)—a mechanism first described in humans by Walker et al. (1961) and later studied intensely in a comparative perspective by Knut Schmidt-Nielsen and collaborators (e.g. Jackson and Schmidt-Nielsen 1964).

**Fig. 1:**
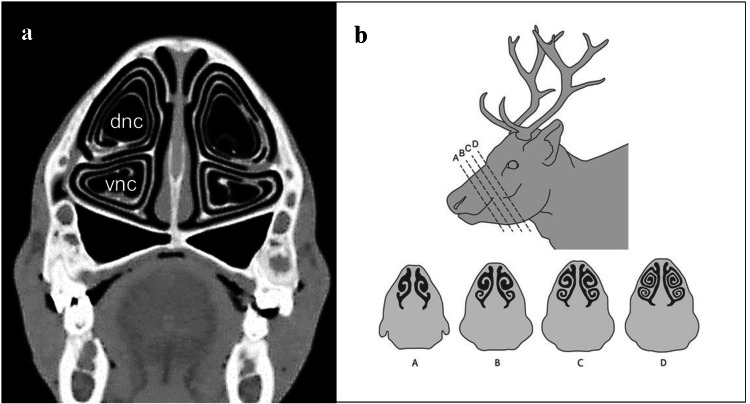
Cross-sectional images of the nasal turbinate structures from the maxilloturbinal region of the nasal cavity of adult reindeer, illustrating the double-scroll organization of the dorsal (dnc) and ventral (vnc) nasal conchae; **a** CT-image (from Casado Barroso 2014); **b** schematic drawing of serial cross section at the indicated positions A–D of the reindeer nose

Reindeer have evolved elaborate nasal turbinate structures (see Fig. 1) that are perfused via a rich and complex vascular network (Johnsen et al. 1985; Johnsen 1988; Casado Barroso 2014). Thermoregulatory control mechanisms (Mercer et al. 1985) allow animals to either conserve or dispose of respiratory evaporative heat (and water) according to their needs (Blix and Johnsen 1983). Turbinates [*conchae nasalis dorsalis, media* and *ventralis*—Nomina anatomica veterinaria (Anonymous 2017)] are structures in the nasal cavity that enhance the surface area of the nasal mucosa that is in direct contact with the respired air stream. Turbinate surfaces, besides being large, are also typically closely arranged, leaving only small air spaces between adjacent surfaces (Negus 1958). Both factors promote efficient transfer of heat and water between air and nasal mucosa (Collins et al. 1971). The three-dimensional design of the turbinate structures is, thus, expected to influence the efficiency of heat and water exchange processes.

We have recently found that the nasal cavity anatomy has substantial effects on its temperature profiles. This was demonstrated after development of a computational model of the reindeer nose by Magnanelli et al. (2017), which allowed simulations to be carried out, with different states and hypothetical designs of the nasal system during respiration. Compared to a hypothetical reference nose with constant geometry and histology along its length, the entropy production profile for the reindeer nose was more uniform and the total entropy production during respiration was lower, at low to intermediate (thermoneutral) ambient temperatures (–30 to 0 °C). It was hypothesized that natural selection may have favoured structural designs that produce more uniform entropy production profiles, as these reduce the total energy dissipation (Magnanelli et al. 2017).

These observations, further suggest that the efficiency of heat exchange processes is also likely affected by animal age/size, given both dimensional and potential structural differences in the turbinates. In this context, we have studied heat exchange processes in newborn reindeer calves. These are born in late spring (April–June), weighing 5–7 kg, but grow fast and are essentially physically mature by the start of their first winter (Timisjärvi et al. 1979; Blix 2005). This study, thus, aims to shed more light on the ontogeny of nasal heat exchange in reindeer and on potential differences in its efficiency as turbinate structures grow and mature. To show this, we have modelled heat transfer processes and entropy production for the newborn reindeer calf and compared model outputs with results for mature conspecific, in both situations assuming the animals to be at rest and in an energy conserving mode in the cold. Furthermore, we have studied the role of some important parameters on heat and mass transfer: respiratory minute volume, friction factor in the nasal cavity and extent of venous vascularization.

The study is in a wide sense motivated by a wish to understand how high energy efficiency is realized in nature, seeking inspiration for engineering design. Nature-inspired chemical engineering is proposed as a discipline (Coppens 2012) useful in a resource-limited world.

## Materials and methods

### Animals

Three newborn calves and three 6–12 months old [physically mature (cf. Timisjärvi et al. 1979)] reindeer were used for anatomical studies of their nasal cavities. Two of the calves and the three mature animals were euthanized for this purpose while the third calf was found still-born. Animals were euthanized with an intravenous overdose of pentobarbital (50 mg per kg body mass of Euthasol^®^ 400 mg/ml; Le Vet. Pharma BV, The Netherlands), as administered via a venoflon catheter in the femoral vein, after prior sedation with xylazine (0.5 mg per kg body mass of Rompun 20 mg/ml; Bayer Animal Health GmbH, Germany) (*n* = 2 calves and *n* = 2 mature animals), or by use of a captive bolt (*n* = 1 mature animal). Dead animals were decapitated and their heads frozen at − 20° for later computer tomography (CT) scanning. Euthanasia was conducted according to the Norwegian Animal Welfare Act and the study was approved by the Norwegian National Animal Research Authority (permit no. 5399).

Physical and physiological parameters for example animals (a mature reindeer and newborn calf) are summarized in Table 1.

**Table 1:** Comparison of physiological and physical parameters for the mature and newborn reindeer, at rest and thermoneutrality

	Mature reindeer	Newborn reindeer	Unit	References
*M*_body_	54	6.67	kg	Casado Barroso (2014)
*T*_body_	38.4	39.5	°C	Markussen et al. (1985) and Soppela et al. (1986)
*V*_min_	0.10	0.25^a^	dm^3^ min^–1^ kg^–1^	Blix and Johnsen (1983)
*f*_*a*_	10	31^a^	breaths min^–1^	Blix and Johnsen (1983)
*L*_nose_	0.2	0.08	M	Casado Barroso (2014)

*M*_*body*_ animal mass, *T*_*body*_ body core temperature, *V*_*min*_ respiratory minute volume, *f*_*a*_ respiratory frequency, *L*_*nose*_ length of the nasal cavity

^a^ Values predicted based on data on resting metabolic rate/oxygen uptake in newborn reindeer (Markussen et al. 1985), assuming similar proportionality between oxygen uptake and *V*min and *fa*, respectively, in newborn as in mature reindeer. Data on resting metabolic rate of mature reindeer from Nilssen et al. (1985)

### Computed tomography scanning

CT-scanning was conducted on frozen reindeer heads using a Siemens Biograph 64 slice PET/CT (Munich, Germany), which generated images of the nasal turbinates with a maximum resolution of 100 μm. CT-scan images were used for determination of the mucosal surface area exposed to the air stream and of air space volumes throughout the nasal cavity (from nostrils and until the pharynx). Measurements of the cross-sectional area and cross-sectional mucosal perimeter of individual slices were made for every tenth slice, each of 0.6 mm thickness, using ImageJ (Rasband, W.S., ImageJ, U. S. National Institutes of Health, Bethesda, Maryland, USA, https://imagej.nih.gov/ij/, 1997–2014). In this context, regions of the nasal cavity that are not directly exposed to the respired air stream (e.g., the mucosa of the innermost ethmoidal turbinates) were not included, since these surfaces were presumed to have little impact on the conditioning of respired air (e.g., Craven et al. 2010). Measurements were taken separately in the right and left nasal passages (Tables 2, 3).

**Table 2:** Parameters used to simulate respiration, from Magnanelli et al. (2017)

	Value	Unit
*φ*_amb_	90	%
*p*_amb_	1.0	Bar
*c*_*p,b*_	4.5	kJ kg^–1^ K^–1^
*ρ*_*b*_	1.0	kg dm^–3^
*k*_*b*_	0.5	J m^–1^ s^–1^ K^–1^
*c*_*p,m*_	4.2	kJ kg^–1^ K^–1^
*ρ*_*m*_	1.0	kg dm^–3^
*k*_*m*_	0.6	J m^–1^ s^–1^ K^–1^
*d*_*m*_	0.7	Mm
*F*_*b*_	0.22	g min^–1^ kg^–1^_animal_

*φ*_*amb*_ ambient air relative humidity, *p*_*amb*_ ambient pressure, *c*_*p,b*_ blood heat capacity, *ρ*_*b*_ blood density, *k*_*b*_ blood thermal conductivity, *c*_*p,m*_ mucus heat capacity, *ρ*_*m*_ mucus density, *k*_*m*_ mucus thermal conductivity, *d*_*m*_ mucus thickness, *F*_*b*_ blood mass flow to the nose

**Table 3:** Average venous cross-section area, *A*_ven_, and perimeter, *γ*_ven_, for five different cross-sections along the nasal cavity of a mature reindeer

*z/L*_nose_ (–)	*A*_ven_ (m^2^)	*N*_ven_/*γ* (cm^–1^)	*A*_ven_/*γ* (m^2^ cm^–1^)	*γ*_ven_ (m)	*γ*_ven_/*γ* (m cm^–1^)
0	5.2E–07	13	6.7E–06	2.5E–03	3.3E–02
0.25	6.1E–07	10	6.1E–06	2.8E–03	2.8E–02
0.5	1.6E–07	12	1.9E–06	1.4E–03	1.7E–02
0.75	2.8E–07	8	2.3E–06	1.9E–03	1.5E–02
1	7.9E–07	7	5.5E–06	3.1E–03	2.2E–02

The number of veins per unit length of mucosal lining, *N*_ven_/*γ*, gives the density of venous area and perimeter along the mucosal lining of a cross-section. Data from Casado Barroso (2014)

In image analyses, the auto-thresholding function was used, which is a variation of the IsoData algorithm. This procedure divides the image into object and background by setting an initial threshold, and then the averages of the pixels at or below the threshold, and pixels above, respectively, are computed. The average of those two values is computed, the threshold is incremented and the process is repeated until the threshold is larger than the composite average (see https://imagej.nih.gov/ij/).

### Theoretical formulation

The thermodynamic description of the nasal system was formulated by Magnanelli et al. (2017). Essential parts are restated here for ease of reading.

### The nasal system

The complex geometry of the reindeer nose is characterized by richly vascularized scroll-like turbinates inside their nasal cavities (Johnsen 1988; Casado Barroso 2014; Magnanelli et al. 2017). The structures are covered by a thin mucosa that produces mucus, which protects the underlying tissues from dehydration. As cold, dry air flows through the nasal cavity during inspiration it is conditioned, i.e. humidified and warmed to deep body temperature (38–39 °C, see Table 1), before it reaches the lungs, using the turbinate mucosa as a heat source. During exhalation, saturated air at body temperature passes over the turbinate surfaces. During heat conservation, these remain cold after the inspiratory phase and subsequently cool down the exhaled air (from the lungs), upon passage, allowing recovery of some of the heat and water that was added upon inhalation. This process is referred to as nasal temporal counter-current heat exchange, or nasal heat exchange, in short (Jackson and Schmidt-Nielsen 1964; Blix and Johnsen 1983; Johnsen et al. 1985; Johnsen 1988).

**Fig. 2:**
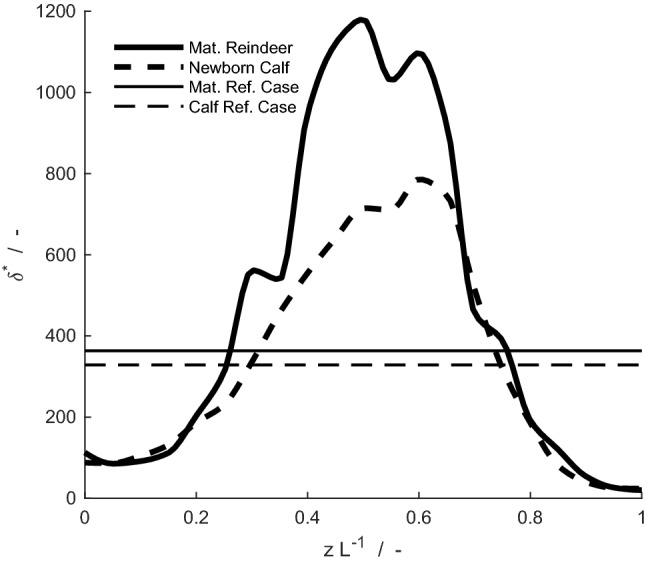
Complexity of nasal cavity illustrated through the dimensionless coefficient, ${\delta }^{*}={\gamma }^{2}/A,$ where *γ* is the nasal mucosa perimeter and *A* is the cross-sectional area, as a function of the scaled position along the nose for the mature reindeer (thick solid line), its reference case (thin solid line), the newborn reindeer (thick dashed line) and its reference case (thin dashed line)

Nasal complexity, given by cross-sectional area, $A$, and perimeter, $\gamma$, for any position, $z$, along the nasal cavity, $0<z<{L}_{\mathrm{n}\mathrm{o}\mathrm{s}\mathrm{e}}$, is effectively visualized through a dimensionless coefficient, ${\delta }^{*}={\gamma }^{2}/A$. The usage of non-dimensional parameters allows their geometric comparison independently of animal size (newborn calf, juvenile or adult). In Fig. 2, the parameter ${\delta }^{*}$ increases/decreases when the geometric complexity of the nasal cavity increases/decreases. Usually the dimensional parameter hydraulic diameter, ${D}_{\mathrm{h}}=4A/ \gamma$, is used for the simplified 1D modelling of the fluid flows through the complex ducts (White 2003).

In this representation of the nasal system, position $0$ is the position of the nostrils and ${L}_{\mathrm{n}\mathrm{o}\mathrm{s}\mathrm{e}}$ is at the start of the juncture of the nasal and oral cavities, the oropharynx. Since systems of different lengths are compared, the spatial domain is scaled to lie between $0$ and $1$.

**Fig. 3:**
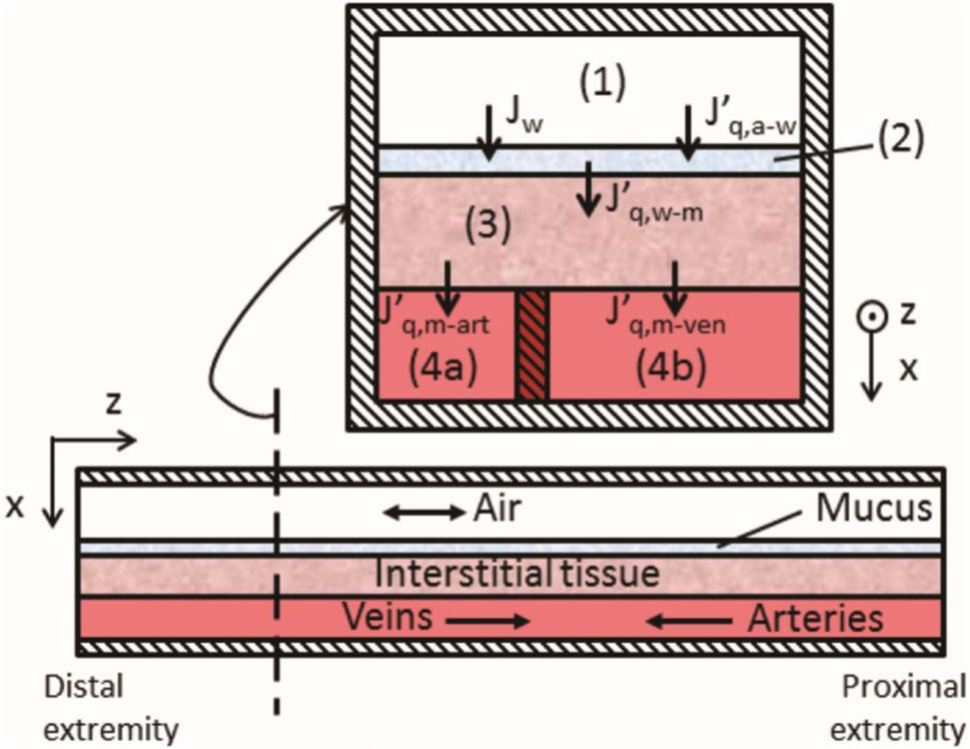
Illustration of interactions between the different subsystems of the animal nose. Air flows from distal extremity (nostrils) to proximal extremity (oropharynx) along the *z*-direction. State variables for subsystems are cross-section averaged and transport between subsystems happen along the *x*-direction. *J*_w_ represents mass flux of water, while *J*’_*q,i-j*_ is the measurable heat flux between subsystems *i* and *j*. Illustration by Magnanelli et al. (2017)

In modelling, the nasal structure was divided into five different subsystems. These subsystems are airway, mucus/liquid layer, interstitial tissue of the mucosa, arteries and veins. An illustration of the theoretical model is presented in Fig. 3. To explain the variation of thermodynamic variables in the nasal system, dynamic one-dimensional mass and energy balances were formulated for each of the subsystems.

Model-based simulations of respiration in reindeer were compared with reference cases to quantify how the nasal anatomy affects the efficiency of heat and water exchange processes. Reference cases are represented by hypothetical noses with similar dimensions but a constant circular geometry and diameter along the nasal cavity, illustrated for the airway subsystem in Fig. 2 by straight lines. These cases are chosen such that the total surface area and total volume of all subsystems of the nose are equal to that of the real animal (newborn and mature reindeer, respectively).

### Assumptions and simplifications

A number of simplifications and assumptions were employed by Magnanelli et al. (2017) to develop the computational model.

- Any variation in the oxygen and carbon dioxide content of the air is ignored, and the molar composition is taken as constant at 79% nitrogen and 21% oxygen.
- Properties of interstitial tissues are approximated by those of blood and are assumed constant in time.
- Blood and blood vessels are assumed to have invariant properties. Any pressure change in blood vessels is ignored.
- Physiological parameters, such as respiratory minute volume and frequency, are based on values found in, or extrapolated from, the literature (see Table 1) and are assumed constant in time and over the ambient temperature range.
- Dynamic variations of subsystem geometries are ignored.
- The theoretical model is one-dimension in space and state variables are cross-section averaged at all points along the length of the nasal cavity.
- Any phase transition from liquid water to ice is unaccounted for. However, condensation/evaporation of water and its relative latent heat is taken into consideration.
- Metabolic heat production in nasal tissues is ignored.
- The air flow is assumed to be turbulent.

The last assumption is not obvious. As far as the human nose is concerned, the literature support both laminar and turbulent flow. The reindeer nose is, however, much more complex and has, as well special features which promote turbulence (Johnsen 1988; Casado Barroso 2014). This supports the assumption. When air flow is at its maximum during inhalation/exhalation, Reynolds numbers are relatively low for the mature reindeer, ranging from 100 to 900. Still, Womersley numbers are above unity for large parts of the nasal cavity. In terms of the ratio of Reynolds to Womersley numbers, airflow in the nasal vestibule and a majority of the maxilloturbinate region fall (ranging from 250 to 500) within a transitional region between laminar and turbulent flow (Craven et al. 2007).

The use of a 1D model to describe a highly three-dimensional system will be a simplification of the system. Computational fluid dynamics (CFD) simulations would allow to take the full three-dimensional geometry into consideration, but at the same time would require a considerable computational effort for the simulation of a dynamic system, where inputs and outputs varies continuously over time. The procedure used here, makes use of an area averaging technique, well established in chemical engineering (Jakobsen 2008).

### The entropy production

The classical formulation of the second law of thermodynamics states:

$$\Delta S+\Delta {S}_{0}\ge 0,$$

where Δ*S* is the change in entropy of the system, and Δ*S*_0_ is the change in entropy of the environment (Kjelstrup and Bedeaux 2008). The sum of entropy changes for an ideal reversible process is zero. In nature, processes are irreversible. Thus, the sum of entropy changes is positive, and takes the name of entropy production (${\Sigma }_{\mathrm{i}\mathrm{r}\mathrm{r}})$. According to the Gouy–Stodola theorem, the energy dissipated due to irreversibility in a process, ${E}_{\mathrm{l}\mathrm{o}\mathrm{s}\mathrm{t}}$, is directly related to the entropy production:

$${E}_{\mathrm{l}\mathrm{o}\mathrm{s}\mathrm{t}}={T}_{\mathrm{a}\mathrm{m}\mathrm{b}}{\Sigma }_{\mathrm{i}\mathrm{r}\mathrm{r},}$$

where ${T}_{\mathrm{a}\mathrm{m}\mathrm{b}}$ is the temperature of the ambient (Gouy 1889). It is, therefore, of interest to study the entropy of the system. A more exhaustive description of the thermodynamic model can be found in Magnanelli et al. (2017).

### Recovery of heat and water during exhalation

During inhalation, water is added to the dry inhaled air from the mucus/liquid layer. The water added, ${M}_{\mathrm{w},\mathrm{a}\mathrm{d}\mathrm{d}\mathrm{e}\mathrm{d}}$, can be calculated from the amount of water that leaves the lungs during exhalation and the amount that enters the nose during inhalation.

$${M}_{\mathrm{w},\mathrm{a}\mathrm{d}\mathrm{d}\mathrm{e}\mathrm{d}}={\int }_{\text{ex}}{\left(-{F}_{w,a}\right)}_{z=L}\mathrm{d}t-{\int }_{\text{in}}{\left({F}_{w,a}\right)}_{z=0}\mathrm{d}t.$$

The amount of water recovered during exhalation, ${M}_{\mathrm{w},\mathrm{r}\mathrm{e}\mathrm{c}\mathrm{o}\mathrm{v}},$ is found from the amount of water leaving the lungs and the amount that exits through the nostrils:

$${M}_{\mathrm{w},\mathrm{r}\mathrm{e}\mathrm{c}\mathrm{o}\mathrm{v}}={\int }_{\text{ex}}{\left(-{F}_{w,a}\right)}_{z=L}\mathrm{d}t-{\int }_{\text{ex}}{\left(-{F}_{w,a}\right)}_{z=0}\mathrm{d}t.$$

Here ${F}_{w,a}$ is the mass flow of water in the air. Subscripts $\mathrm{e}\mathrm{x}$ and $\mathrm{i}\mathrm{n}$ refer to the exhalation and inhalation parts of the breathing cycle, respectively. The difference between the amount of water added to and recovered from the humid air then gives the amount of water lost to the environment.

The heat added to the air to warm it up during inhalation and the latent heat added due to evaporation of water gives the total heat added to the inhaled air, ${Q}_{\mathrm{a}\mathrm{d}\mathrm{d}\mathrm{e}\mathrm{d}}$:

$${Q}_{\mathrm{a}\mathrm{d}\mathrm{d}\mathrm{e}\mathrm{d}}={\int }_{\text{in}}{F}_{a}{C}_{p,a}\left({T}_{\mathrm{b}\mathrm{o}\mathrm{d}\mathrm{y}}-{T}_{\mathrm{a}\mathrm{m}\mathrm{b}}\right)\mathrm{d}t+{M}_{\mathrm{w},\mathrm{a}\mathrm{d}\mathrm{d}\mathrm{e}\mathrm{d}}{h}_{\mathrm{l}\mathrm{a}\mathrm{t}},$$

where ${F}_{a}$ is the mass flow of humid air, ${C}_{p,a}$ is the heat capacity of the air, and ${h}_{\mathrm{l}\mathrm{a}\mathrm{t}}$ is the latent heat of evaporation of water. The heat removed from the air stream during exhalation and the heat released due to condensation of water gives the total recovered heat, ${Q}_{\mathrm{r}\mathrm{e}\mathrm{c}\mathrm{o}\mathrm{v}}$:

$${Q}_{\mathrm{r}\mathrm{e}\mathrm{c}\mathrm{o}\mathrm{v}}={\int }_{\text{ex}}{F}_{a}{C}_{p,a}\left({T}_{\mathrm{b}\mathrm{o}\mathrm{d}\mathrm{y}}-{T}_{\mathrm{e}\mathrm{x}}\right)\mathrm{d}t+{M}_{\mathrm{w},\mathrm{r}\mathrm{e}\mathrm{c}\mathrm{o}\mathrm{v}}{h}_{\mathrm{l}\mathrm{a}\mathrm{t}},$$

where ${T}_{\mathrm{e}\mathrm{x}}$ is the temperature of the air exiting the nostrils. We find the heat lost to the environment, ${Q}_{\mathrm{l}\mathrm{o}\mathrm{s}\mathrm{t}}$, from the difference between the heat added to the air and the heat recovered.

### The respiratory minute volume

The basic model assumes that example animals were at rest in a thermoneutral environment (Table 1). When the model was run assuming ambient temperatures below thermoneutrality, this would affect respiratory minute volume, *V*_min_, since thermogenetic metabolic processes would then be initiated, which require an increased *V*_min_ (Blix and Johnsen 1983). During inhalation, the consequence would be that a larger volume of air must be warmed per minute. We, therefore, studied the model’s sensitivity to respiratory minute volume, friction factor and nasal vascularization.

The dry air flow, *F*_dry_, at any time, *t*, during a breathing cycle of duration *τ*_breath_ (calculated from the respiratory frequency, *f*_a_) is modelled as a sinusoidal function with no resting phase between inhalation and exhalation (Magnanelli et al. 2017).

$${F}_{\mathrm{d}\mathrm{r}\mathrm{y}}={F}_{\mathrm{d}\mathrm{r}\mathrm{y},\mathrm{m}\mathrm{a}\mathrm{x}}\mathrm{s}\mathrm{i}\mathrm{n}\left(\frac{2\uppi }{{\uptau }_{\mathrm{b}\mathrm{r}\mathrm{e}\mathrm{a}\mathrm{t}\mathrm{h}}}t\right)$$

The maximum dry air flow during the breathing cycle, *F*_dry,max_, is calculated from the respiratory minute volume. Increasing the respiratory minute volume leads to larger air flow, and significantly affects the state of the various nasal subsystems.

### The nasal friction factor

While the pressure drop due to frictional flow is negligible, typically lower than 300 Pa during calm breathing (Magnanelli et al. 2017), the friction factor has a significant effect on convective heat transfer. For instance, when the friction factor increases, the resistance to convective heat transfer decreases. An empiric expression for the friction factor for flow through the nose of a reindeer has yet to be established. However, an expression for flow through the human nasal cavity for similar respiratory minute volumes is available (Zamankhan et al. 2006).

$$f=\frac{47.78}{\text{Re}}\left(1+0.127{\text{Re}}^{0.489}\right).$$

Here Re is the Reynolds number and *f* is the friction factor. However, reindeer nasal structure is more complex and convoluted than that of humans and it is, therefore, likely that such a structure would lead to a larger friction factor than the human one. We will, therefore, further investigate how a higher friction factor would influence respiration. We do this by comparing the results obtained with the friction factor calculated according to the equation above with those obtained with an increased friction factor.

A complete description of how heat and mass transport coefficients are affected by the friction factor and by other parameters can be found in Magnanelli et al. (2017).

### Nasal vascularization

In a study of the nasal anatomy of reindeer, it was noted that the cross-sectional area of veins was some 6–10 times larger than that of arteries (Casado Barroso 2014). In the theoretical model, blood flows through arteries and veins in a counter-current configuration. A portion of the amount of heat carried by arterial blood is transferred to venous blood, sending warmed venous blood back to the body core (Johnsen et al. 1985). To investigate the importance of the abundant venous supply, we ran a simulation with the density of veins reduced by a factor of three. Thereby, venous blood velocity was doubled, but, more importantly, the geometrical alteration decreased the interfacial area between veins and interstitial tissues at any point along the nasal cavity, also by a factor of three.

## Results

**Fig. 4:**
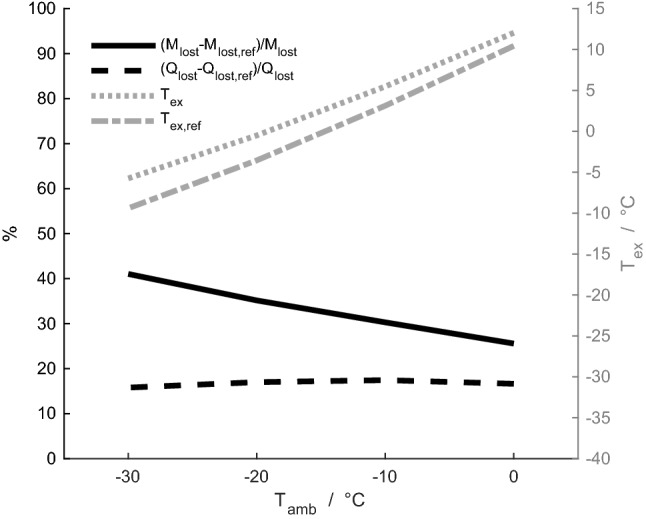
Mass-averaged temperature of exhaled air, *T*_ex_, for the newborn reindeer (grey dotted line) and its reference case (grey dash-dotted line), and percentage difference between the newborn reindeer calf and its reference case for the heat lost, *Q*_lost_ (black dashed line), and mass of water lost, *M*_lost_ (black solid line), to the environment

**Fig. 5:**
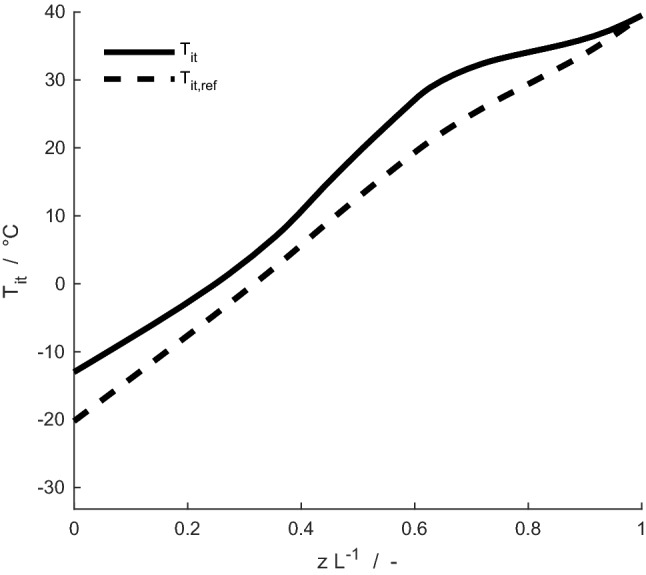
Time-averaged temperature profiles of interstitial tissues, *T*_it_, for the newborn reindeer calf (solid line) and its reference case (dashed line) at an ambient temperature of – 30 °C

**Fig. 6:**
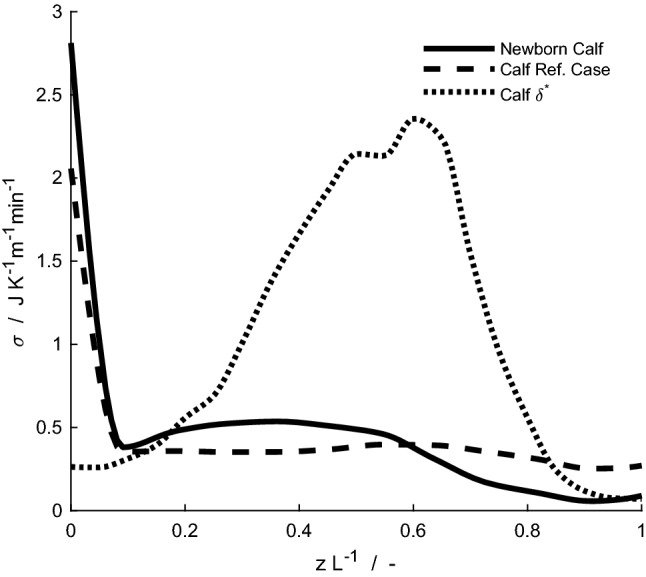
Local entropy production during respiration, σ, for the newborn reindeer calf (solid line) and its reference case (dashed line) at an ambient temperature of – 30 °C. Nasal complexity for the calf included (dotted line), ${\delta }^{\mathrm{*}}={\gamma }^{2}/A$ where *γ* is the nasal mucosa perimeter and *A* is the cross-sectional area

**Fig. 7:**
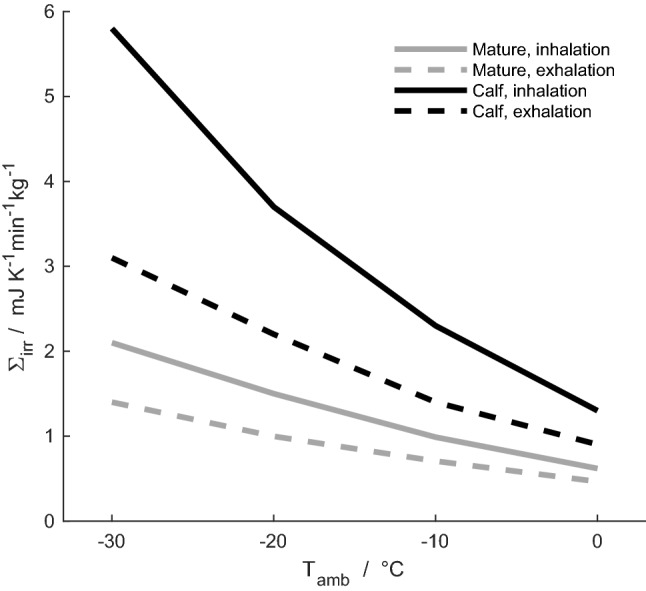
Specific total entropy production, *Σ*_irr_, during inhalation (solid lines) and exhalation (dashed lines) for the mature reindeer (grey lines) and the newborn reindeer calf (black lines), for ambient temperatures of – 30 °C, – 20 °C, – 10 °C and 0 °C

**Fig. 8:**
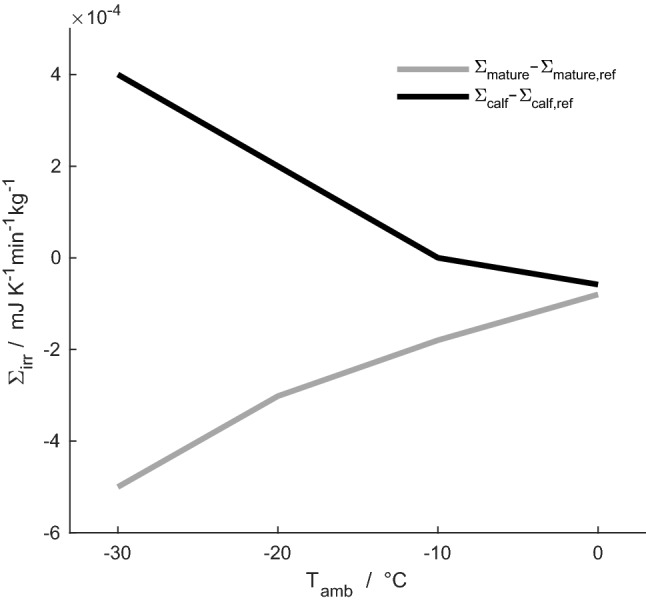
Difference in specific total entropy production, *Σ*_irr_, between newborn calf (black line) and mature reindeer (grey line) and their respective reference case, for ambient temperatures of –30 °C, –20 °C, –10 °C and 0 °C

**Fig. 9:**
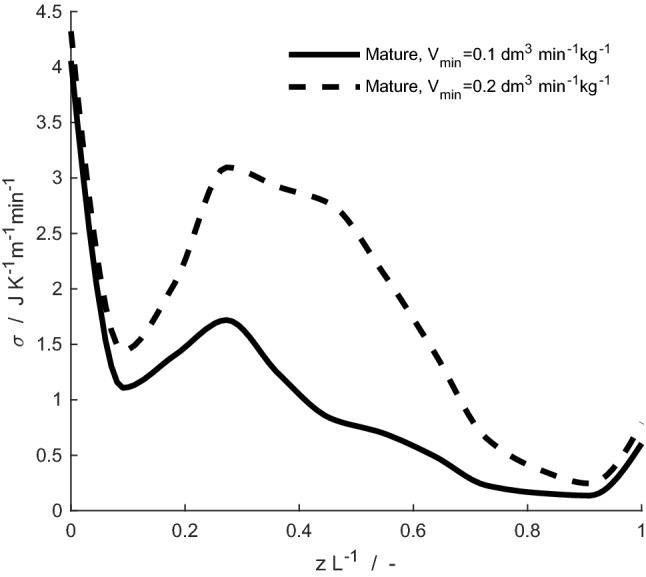
Local entropy production, *σ*, during respiration for the mature reindeer with the original respiratory minute volume (solid line), *V*_min_, and doubled (dashed line), for an ambient temperature of – 30 °C

**Fig. 10:**
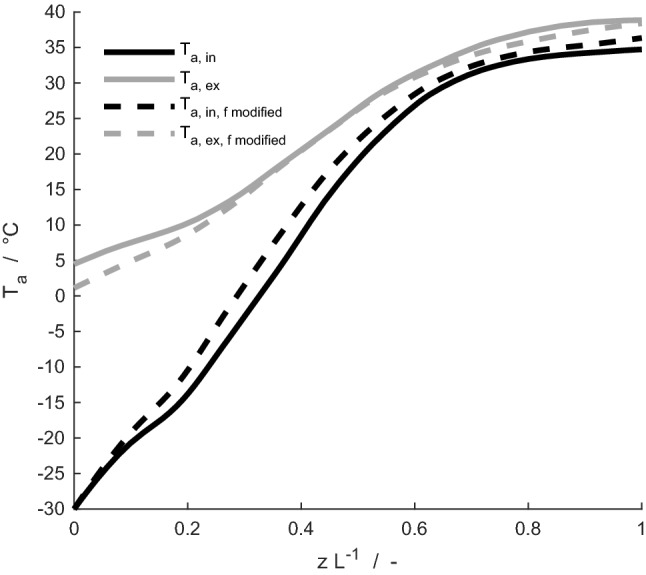
Mass flow-averaged temperature, *T*_a_, of inhaled (black lines) and exhaled air (grey lines) for the mature reindeer with the original friction factor (solid lines), and a case where the exponent in the friction factor expression was doubled (dashed lines), for an ambient temperature of – 30 °C

**Fig. 11:**
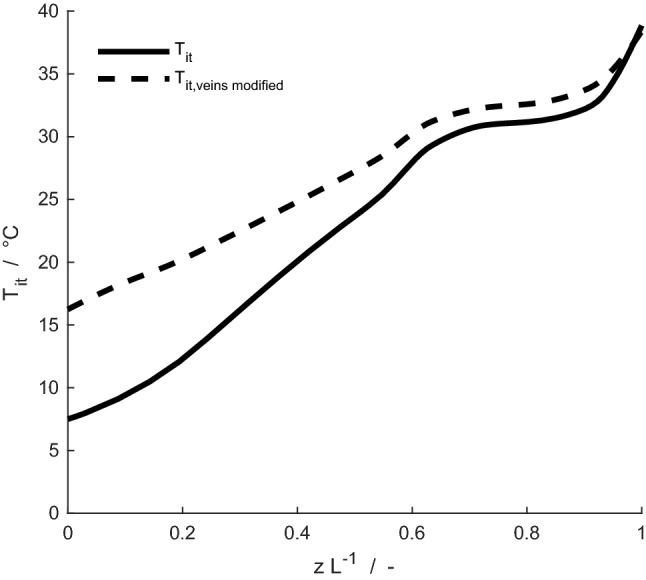
Time-averaged temperature of interstitial tissues, *T*_it_, for a mature reindeer with the original geometry of veins (solid line), and the case where the density of veins in the nasal mucosa is divided by a factor of three (dashed line), for an ambient temperature of – 30 °C

The results are shown in Figs. 4, 5, 6, 7, 8, 9, 10 and 11. We have simulated respiration for the newborn reindeer and compared the results with those from the mature animal. Specifically, we have investigated the effect on the system by varying a selection of physiological parameters relevant to respiration, i.e. the respiratory minute volume, nasal friction factor and geometry of veins, in all cross sections along the nasal cavity. To allow comparison, we have partially run the model at very low ambient temperatures, although these are unlikely encountered by newborn reindeer.

### Heat and water losses to the environment during respiration in the newborn calf

The mass-averaged temperature of exhaled air, as well as the difference between the newborn reindeer and its reference case (i.e., a hypothetical nose with similar dimensions but a constant circular geometry and diameter along the nasal cavity) in terms of heat and water losses to the environment, are presented in Fig. 4. In the ambient temperature range 0 to – 30 °C, the model predicts that the newborn loses 26–41% more water to the environment than does its reference case, and it loses 16–17% more heat. The mass-averaged temperature of exhaled air is, for the newborn, 1.6–3.6 °C higher than for its reference case, for the considered ambient temperature interval. For an ambient temperature of – 30 °C, the time-averaged temperature profile of interstitial mucosal tissues is shown for the new-born calf and its reference in Fig. 5. Along the whole nasal cavity of the calf, its interstitial mucosal tissues are on average warmer than for the reference. The difference is in the range of 0.2–7.9 °C, and for tissues around the nasal orifice the difference is roughly 7 °C.

### Entropy production during respiration in the newborn calf

The local entropy production profile during respiration at an ambient temperature of – 30 °C is shown for the reindeer calf and its reference case in Fig. 6. In terms of total entropy production, we find 0.0019 J K^–1^ cycle^–1^ (i.e. for a full inspiration and expiration cycle) for the calf and 0.0018 J K^–1^ cycle^–1^ for its reference case. The nasal anatomy of the reindeer calf gives a 5% increase in total entropy production in simulated respiration compared to the uniform anatomy of its reference case. The specific total entropy production during respiration, at ambient temperatures of – 30 to 0 °C, is shown for the mature reindeer and the newborn in Fig. 7. It is 1.9–2.8 times larger for the newborn than for the mature animal in this temperature range. The specific entropy production during inhalation is 1.4–2 times larger than during exhalation for both specimens. Absolute differences in specific total entropy production during respiration between animals and their respective reference cases are presented in Fig. 8. Negative values indicate that the animal performs better than its reference case in terms of the entropy production (which, hence, is lower for the newborn than for the reference case). For the ambient temperature of 0 °C, both the mature and the newborn reindeer have lower specific total entropy production than their respective reference cases. At temperatures below 0 °C, the differences are – 10% to –15% for the mature reindeer and – 1% to 4% for the newborn, compared to their respective reference cases, i.e. the difference is positive for – 20 and – 30 °C. Thus, our model predicts that at ambient temperatures of – 20 °C and lower, the newborn would be unable to warm up the air to within 6 °C of its body temperature. At ambient temperatures of – 10 and 0 °C, 90% and 70% of the length of the newborn's nose are required to warm up the air.

### Effects of varying respiratory minute volume in the mature reindeer

The effect of doubling the minute volume for the mature animal, (i.e. from 0.1 to 0.2 dm^3^ min^–1^ kg^–1^, corresponding to an increase in the volumetric flow rate from 5.4 to 10.8 dm^3^ min^–1^) is illustrated in terms of local entropy production in Fig. 9. A doubling of the minute volume leads to a 91% increase in the total entropy production (from 0.0187 to 0.0358 J K^–1^ cycle^–1^). The total entropy production is smaller for the mature reindeer than for its reference case also when the respiratory minute volume is doubled. The amount of heat and water lost to the environment increases, but not proportionally, from 267 J min^–1^ and 1.4 mg min^–1^ to 582 J min^–1^ and 2.5 mg min^–1^, respectively. To warm up the inhaled air to within 6 °C of the animal's core body temperature in simulations, the mature reindeer needs 74% of the length of its nose when the ambient temperature is – 30 °C. Whereas when the minute volume is doubled, the reindeer needs 97% of the nasal length to warm up the air. In simulations of respiration, the average temperature of all subsystems is lower when the minute volume increases, due to the increased need for air warming.

### Effects of varying nasal friction factor in the mature reindeer

Doubling the friction factor leads to a 1.5–2 times larger Reynolds number range. The Reynolds number has a direct influence on the heat and mass transfer in the nasal cavity, as larger Reynolds number reflects higher turbulence inside the nasal cavity (Incropera et al. 2007). The effect on the average temperature profile of air along the nasal cavity at an ambient temperature of – 30 °C, is shown in Fig. 10. The air temperature is for large parts of the nasal cavity 2–3 °C higher during inhalation, compared to before doubling of the friction factor. During exhalation in an ambient temperature of – 30 °C, the temperature of air exiting the nostrils is approximately 3 °C lower when the friction factor is doubled. For a breathing cycle, a decrease from 0.0187 to 0.0173 J K^–1^ cycle^–1^, a 7% decrease, is observed in the total entropy production. The doubled friction factor leads to a decrease in the amount of heat and water lost, from 267 J min^–1^ and 1.4 mg min^–1^ to 242 J min^–1^ and 1.2 mg min^–1^, respectively.

### Effects of varying branching of veins along the nasal cavity of the mature reindeer

Figure 11 shows the average temperature profile of interstitial tissues (the mucosa) for the mature reindeer when relating to the actual cross-sectional proportion of veins vs. arteries (see Casado Barroso 2014), and for a simulation where the density of veins has been reduced (by a factor of three). The average temperature of interstitial tissues is higher when venous density is reduced, with the largest difference between the two cases being roughly 9 °C in tissues near nostrils. The heat and water losses increase, from 267 J min^–1^ and 1.4 mg min^–1^ to 303 J min^–1^ and 2.2 mg min^–1^, respectively.

## Discussion

Our simulations demonstrate that the nasal anatomy and its structural design have a profound effect on nasal heat and water exchange and entropy production during respiration, in both the mature and the newborn reindeer. These simulation results were compared with hypothetical reference noses, which have the same total volumes and surface areas as the animals' nasal subsystems, but where the reference nose anatomy is assumed to be uniform over the nasal cavity. In this manner, we investigated the impact of the nose structure. Simulations of a mature reindeer were investigated before in a similar manner by Magnanelli et al. (2017), who showed that, compared to a reference case defined as above, the reindeer nasal anatomy gave a 9–20% reduction in total entropy production. This occurred with roughly the same heat and water losses to the environment, while the temperature of interstitial mucosal tissues was kept warmer (Magnanelli et al. 2017). When comparing respiration of the newborn reindeer with its reference case, however, the newborn reindeer nose appears to perform worse in terms of specific total entropy production and heat and water losses to the environment (see Figs. 4, 5, 6).

Thus, heat and water losses to the environment during simulated respiration were significantly higher for the newborn than for its reference, particularly at low ambient temperatures. The losses are related to the exhaled air temperature, in the sense that the lower exhaled air temperature of its reference case than that of the newborn means that less heat was lost, and a larger amount of heat was recovered, during exhalation for the reference case. In terms of minimizing losses to the environment, it is beneficial to keep a cold mucosal lining, such that the driving forces for heat and mass recovery during exhalation becomes larger. For – 30 °C, the model predicts sub-zero tissue temperatures near nostrils (Fig. 5), indicating that extra heat supply to this region may be necessary to prevent tissue freezing, as previously recognized from experimental studies in mature reindeer (Johnsen et al. 1985).

These experiments showed that at an ambient temperature of – 30 °C, the turbinates, i.e. the middle section of the nasal cavity, held an average temperature 10–15 °C lower than that of the tissues near the nasal orifice. It was hypothesised that the warm distal tissues were a result of blood perfusion independent of the nasal cavity tissues, presumably to prevent freezing injuries at very low ambient temperatures (Johnsen et al. 1985). Magnanelli et al*.* (2017) compared these experimental values with the temperature profile predicted for a mature reindeer using the present model, noting also the model’s inability to accurately predict the temperature of tissues near the nasal orifice, since an independent perfusion source of the nasal orifice was not taken into account. Experiments have yet to be conducted to accurately determine blood flow in absolute numbers in the nostril region of reindeer (both newborn and mature). The simulations for the newborn that predicted sub-zero temperatures for these tissues at an ambient temperature of – 30 °C, support the idea that independent perfusion is indeed important in keeping the most peripheral tissues from freezing, should calves ever be exposed to such extreme temperatures.

Magnanelli et al. (2017) found that the complex nasal anatomy of the reindeer gave a more uniform entropy production profile than the less complex, straight reference nose, whereas tissues were on average warmer, despite similar amounts of heat and water lost to the environment. It is known that a uniform distribution of entropy production is a characteristic of systems with high energy efficiency (Kjelstrup et al. 2018). Optimization studies of industrial equipment have also shown that energy-efficient processes are characterized by uniform entropy production profiles (Johannessen and Kjelstrup 2005; Kjelstrup and Bedeaux 2008; Wilhelmsen et al. 2010). Gheorghiu et al. (2005) showed, using a theoretical model for mass transport in the mammalian lung that the fractal-like architecture of the lung gave uniform entropy production across all branches of the bronchial tree and ensured at the same time optimal energy-efficient functioning. We find these similarities striking, suggesting that the structures that have developed and are found in nature, could be of general interest in engineering. Some first attempts in this direction have already been made, where the geometry of a plug-flow reactor was numerically optimized to reduce entropy production (Magnanelli et al. 2019).

For the ambient temperature of – 30 °C, the local entropy production profile for the newborn may be described as less uniform than in its reference case (Fig. 6), and the total entropy production is indeed 5% higher. In the anatomical study of the newborn and mature reindeer, it was found that the nasal surface-area-to-mass ratio was large for the newborn (i.e. 40.79 cm^2^ kg^–1^ compared to 14.70 cm^2^ kg^–1^ for the mature animal). These numbers alone may suggest that the necessary anatomy for energy-efficient nasal heat exchange is already present from birth (Casado Barroso 2014). In contrast, the present computational model allows us to hypothesise that the calf at birth has a nasal anatomy that is not as energy efficient as that of the adult, and that the mature efficiency developed during the first months of its life.

As it grows older during the summer, the nasal structure becomes more efficient from an energy conservation perspective. The development (see above) prepares the animal to face the coming arctic winter, at which time the animal must be self-sufficient. In the short periods of cold weather during late spring and summer, when the animal is still a calf, the immaturity of the nasal structure may not be so critical. Fast growth may be more important than energy efficiency, particularly since feed, available as milk via its mother, usually is not a limiting resource.

The less complex structure of the nasal cavity (and the resulting smaller nasal surface area) near the nostrils helps shifting heat and water exchange processes away from the nostril region and into the complex turbinates. Considering the local entropy profile for the mature animal (Fig. 9), doubling the respiratory minute volume does not significantly increase the energy dissipation near the nostrils. Instead, air quickly flows through the nostril region, and a maximum is observed in the local entropy production around the region where complex turbinate structures greatly increase the nasal surface area. Local entropy production profiles for the reindeer are, therefore, more uniformly distributed over the nasal cavity, than for the reference case. Doubling the respiratory minute volume leads to roughly a doubling of total entropy production and heat and water losses during respiration, if other physiological parameters (e.g., nasal blood flow) are kept constant.

It is likely that the friction factor for air flow through the reindeer's complex nasal cavity is considerably higher than values given by correlations for flow in the nasal cavity of humans (Zamankhan et al. 2006) and used in this work. In terms of geometry, the human nasal cavity is considerably simpler than that of the reindeer, featuring an almost open channel for air flow. Thus, the simulations with increased friction factor might be more relevant for the description of the reindeer respiration processes than the reference ones. The friction factor has a large influence on the convective heat transfer between air and mucus layer. A high friction factor, causing higher convective heat transport, would lead to a further reduction in heat and water losses as well as in total entropy production.

By reducing the venous density in the mucosal lining by a factor of three, we aimed to elucidate the effect of the extent of venous vascularization on the heat exchange processes in the nose. Near the nostrils, the average temperature of tissues is 10 °C higher when venous vascularization is reduced. By investigating the heat flux from interstitial tissues to veins along the nasal cavity, we find that for a breathing cycle, the heat transfer is reduced from 141 to 87 J cycle^–1^ with less vascularization. The abundance of veins compared to arteries may facilitate venous transport of heat back to the body core. This effect is also reflected in terms of local entropy production. Due to the higher temperature of nasal subsystems with reduced venous representation, the heat exchange processes would be shifted toward the nostrils. As a result, the local entropy production profile is less uniform over the length of the nasal cavity, leading to a 6% increase in total entropy production during respiration. Additionally, the amount of heat and water lost to the environment increased. Our simulations, thus, suggest that the abundance of veins may aid in properly controlling temperature profiles in the nose, such that the nose is sufficiently warm, but not excessively warm, keeping the dissipation of heat within limits.

Another physiological parameter of significance to nasal heat and water exchange is the dynamic ability to alternate the blood flow pattern through the nasal mucosa, from counter-current to unidirectional flow. This change decreases the nasal temperature gradient, from proximal to distal part of the nose, thereby allowing for increased energy dissipation in potentially hyperthermic animals. It is achieved by primarily draining venous blood from the distal part of the nasal mucosa via the dorsal nasal (Johnsen et al. 1985). The current computational model only simulates counter-current blood flow. A vascular configuration yielding unidirectional flow could be explored in future simulations of respiration.

In summary, we have continued our studies of arctic animals, with the aim to elucidate the energy efficiency of their respiratory tracts, and the role of the tissue structure for optimal function. We have confirmed previous studies in mature reindeer; that the convoluted structure improves the animal ability to control its energy loss (e.g. Blix and Johnsen 1983; Magnanelli et al. 2017). The role of the venous system in the regulation of the overall nasal temperature profile, ensuring high energy recovery, was pointed out. These findings are important because they point at geometrical structure as an important parameter in energy efficient design. Knowledge of structure–function relationships in the context of energy may benefit man-made creations.
